# Deadliness of Traumatic Subdural Hematomas in the First Quarter of the Year: A Measurement by the American College of Surgeons-National Surgical Quality Improvement Program (ACS-NSQIP)

**DOI:** 10.7759/cureus.50860

**Published:** 2023-12-20

**Authors:** Rose Fluss, Jessica Ryvlin, Sharon Lam, Muhammad Abdullah, David J Altschul

**Affiliations:** 1 Neurological Surgery, Montefiore Medical Center, Bronx, USA; 2 Neurological Surgery, Albert Einstein College of Medicine, Bronx, USA

**Keywords:** nsqip, season, winter, trauma, subdural hemorrhage, subdual hematoma

## Abstract

Background

Traumatic acute subdural hematoma (ASDH) is a surgical emergency and has been associated with high morbidity and mortality. However, it is not known whether mortality from ASDH occurs more frequently in a particular season.

Methodology

We queried the American College of Surgeons-National Surgical Quality Improvement Program (ACS-NSQIP) from 2016 to 2019. They were identified in the NSQIP using the International Classification of Diseases (ICD-10) code S06.5 to capture all admissions with a primary diagnosis of traumatic subdural hematoma. Mortality rates were reviewed per season, defined as three consecutive months in the year. Demographics such as age, race, ethnicity, height, and weight were reviewed. Comorbidities such as diabetes, risk factors, including smoking history, and hospitalization characteristics, such as admission year, operation year, and inpatient/outpatient treatment type, were also reviewed.

Results

A total of 1,656 patients were included in this study. The mean age of all participants was 70.6 years, with 37% (604/1,656) being female. The mortality rate was highest in January, February, and March at 24.5% (104/425, *P *= 0.045) of admitted patients compared to mortality rates of 18.8% (70/373) in April to June, 18.4% (81/441) in July to September, and 17.5% (73/417) in October to December.

Conclusions

Mortality is significantly greater during the winter months of January, February, and March among patients with ASDH. Despite better survival rates of ASDH over the past two decades, postoperative mortality rates still remain high.

## Introduction

Traumatic acute subdural hematoma (ASDH) is one of the most common neurosurgical emergencies [1,2] and is often associated with high morbidity and mortality [3-6]. Even with improved neurointensive treatment leading to increased survival over the last few decades [7], ASDH remains one of the most lethal of all head injuries and has resulted in increased national adjusted healthcare costs within recent decades [1]. Predictors of mortality associated with ASDH include increased age, injury due to motor vehicle collision, and lower preoperative Glasgow Coma Score [8,9]. However, it remains unclear whether hospital mortality due to ASDH occurs more frequently in certain months. This study aimed to determine which season, if any, had the highest incidence of mortality in patients with ASDH. Data on mortality provide hospitals with an analysis for hospital resource allotment. These trends can offer invaluable information on asset allocation and staff availability planning. Wintertime, for example, has previously been associated with higher mortality rates from cardiovascular and respiratory diseases. It is important to understand the ASDH census to appropriately anticipate increased hospitalizations and mortality and to better treatment protocols. Previous reports have commented on the seasonality of traumatic brain injury (TBI), but this is the first report to our knowledge to comment on season-related mortality peaks.

## Materials and methods

Study design

Data were obtained from the American College of Surgeons National Surgical Quality Improvement Program (ACS-NSQIP) database. A post hoc analysis of the ACS-NSQIP database was then undertaken using a case series design. NSQIP compiles data from randomly selected surgical patients at more than 370 institutions in the United States. This includes preoperative risk factors, variables about patients’ operations, and data on mortality and surgical complications up to 30 days after the operation. NSQIP has trained nurses to collect data from medical records, operative reports, and patient interviews. Detailed documentation regarding recruitment of study participants, study design, and measurement of study data is available on the ACS NSQIP website [10].

Eligible participants for this study underwent craniotomy or craniectomy procedures for subdural hematoma (SDH) between 2016 and 2019. They were identified in NSQIP based on the International Classification of Diseases (ICD-10) code S06.5. Our study population comprised 1,656 patients (Table 1). Patients with the ICD-10 code, S06.5, and all subsequent modifiers were included. Data collected included baseline demographics (such as age, race, ethnicity, height, and weight), medical comorbidities (such as diabetes), risk factors (such as smoking history), and additional hospitalization characteristics (such as admission year, operation year, and inpatient/outpatient treatment).

**Table 1:** The demographic and clinical characteristics of study participants. IQR, interquartile range

	Participants
N	1,656
Age (years), mean	70.6
Sex, *n* (%)	
Female	604 (36.5)
Male	1052 (63.5)
Race, *n* (%)	
White	1,105 (66.7)
Asian	102 (6.2)
Black/African American	193 (11.7)
American Indian or Alaska Native	20 (1.2)
Native Hawaiian or Pacific Islander	7 (0.4)
Unknown/Not reported	229 (13.8)
Hispanic, *n* (%)	
Yes	198 (12.0)
No	1,330 (80.3)
Unknown	128 (7.7)
Treatment setting, *n* (%)	
Inpatient	1,654 (99.9)
Outpatient	2 (0.1)
Admission year, median (IQR)	2018 (2017-2019)
Operation year, median (IQR)	2018 (2017-2019)
Height, median (IQR)	66 (63-69)
Weight, median (IQR)	164 (140-191)
Diabetes comorbidity, *n* (%)	
Yes, insulin	146 (8.8)
Yes, non-insulin	246 (14.9)
No	1,264 (76.3)
Smoking history, *n* (%)	
Yes	263 (15.9)
No	1,393 (84.1)

Outcome measures

The primary outcome measure of interest was the incidence of mortality at 30 days among hospitalization admissions due to ASDH. Patients were divided into four quarters: those who had died from ASDH in January, February, or March (Quarter 1); those who had died from ASDH in April, May, or June (Quarter 2); those who had died from ASDH in July, August, or September (Quarter 3); and those who had died from ASDH in October, November, or December (Quarter 4).

Statistical analysis

The incidence of mortality from ASDH was compared between the four quarters. χ2 analyses were used. Statistical significance was determined by a right-tailed α of 0.05. All data analysis was performed using R version 3.4.2 (The R Foundation for Statistical Computing, Vienna, Austria).

## Results

Demographics 

Characteristics of participants are shown in Table 1. The mean (±SD) age of all participants was 70.6 ± 14, and 36.5% (604/1,656) were female. Demographic results of White, Asian, Black/African American, American Indian or Alaska Native, Native Hawaiian, or Pacific Islander patients were 66.7% (1105/1,656), 6.2% (102/1,656), 11.7% (193/1,656), 1.2% (20/1,656), and 0.4% (7/1,656), respectively. One hundred ninety-eight patients (12.0%) were Hispanic. The median height was 66.0 inches, and the median weight was 164.0 pounds; 23.7% (392/1,656) had diabetes, and 15.9% (263/1656) were either current or former smokers (Table 1).

Mortality

The greatest occurrence of mortality per quarter (104/425, 24.5%) of patients from ASDH occurred in January, February, and March (Quarter 1) (*P *= 0.045). Seventy (70/373, 18.8%) patients died from ASDH in April, May, and June (Quarter 2). Eighty-one (81/441, 18.4%) patients died from ASDH in July, August, and September (Quarter 2). Seventy-three (73/417, 17.5%) patients died from ASDH in October, November, and December (Quarter 4).

In an attempt to identify any potential confounding variables, a chi-square test was performed. The distribution of available demographic variables among patients across the various admission quarters was analyzed using a series of chi-square tests. The demographics were not found to be different in a statistically significant fashion for the following variables: age (*P *= 0.984), sex (*P* = 0.557), race (*P *= 0.369), Hispanic ethnicity (*P *= 0.551), smoking history (*P *= 0.691), and body mass index (BMI; *P* = 0.886). The distribution of diabetes status (including types 1 and 2) among patients was statistically significantly different over the four quarters (*P* = 0.028) (Table 2).

**Table 2:** Chi-square analysis of patients across all quarters. BMI, body mass index

Variable	*χ*^2^	*P*-value
Age	168.64	0.984
Sex	2.075	0.557
Race	16.196	0.369
Hispanic	4.947	0.551
Diabetes	14.199	0.028
Smoker	1.464	0.691
BMI	756	0.886

## Discussion

The results from this retrospective review using ACS-NSQIP provide evidence that there is seasonal variation in in-hospital mortality rates for traumatic ASDH. Our data suggest that mortality in patients admitted due to ASDH was highest in winter months, including January, February, and March (104/425, 24.5%, *P *= 0.045). Mortality for traumatic ASDH patients occurring in other months occurred less frequently but remains dismal at rates ranging from 17.5% to 18.8%.

Using a chi-square test, we failed to reject the null hypothesis that the patient populations were significantly different across admission quarters for age (*P *= 0.984), sex (*P* = 0.557), race (*P *= 0.369), Hispanic ethnicity (*P* =0.551), smoking history (*P *= 0.691), or BMI (*P *= 0.886). Each of the study sets in the four quarters was, therefore, comparable, making the quarters of the year an independent factor. We did find statistical significance for differences in the distribution of the various diabetes classifications (*P *= 0.028). Further investigation may be warranted; however, it is beyond the scope of this manuscript. Unfortunately, more specific diagnostic measures of trauma severity (e.g., Glasgow coma score) were not available in the dataset.

The results published in this report, however, were better than the range of mortality rates reported in the literature for traumatic ASDH. Table 3 lists several reports published in the last decade, along with the number of patients, average age, and reported mortality. Koç et al. reported 60% mortality for patients with ASDH who required surgery [3]. Hatashita et al. found an overall mortality of 55% among surgically treated patients with ASDH [5]. In a review of 144 cases of ASDH from closed head injuries, Fell et al. reported a mortality rate of 48% for those treated within 24 hours of injury and 45% for those treated within 72 hours [11]. Mortality rates among younger patients (age < 65 years) were lower at 18% to 21% [12,13], while the elderly population reported a higher risk for mortality after surgery [5,14]. In a study by Akbik et al., 34% (*n *= 24) of patients above 65 years of age died after craniotomy for evacuation of an ASDH. Mortality rate increased to 44% (*n *= 27) at three months follow-up. Despite a significant improvement in survival over the last 20 years after surgery for ASDH [15], morbidity and mortality surrounding ASDH remain high. A greater understanding of the factors associated with mortality from ASDH is, therefore, warranted.

**Table 3:** Review of mortality rates in acute subdural hematoma in the last decade.

Study	Country	Number of patients	Average age (years)	Mortality (%)
Akbik et al. [4]	United States	62	78	44 (3 months)
Atalay et al. [16]	Turkey	100	42	34 (during hospitalization)
Benko et al. [8]	United States	455	62	7.5 (during hospitalization)
Hsieh et al. [17]	Taiwan	444	63	15 (during hospitalization)
Raj et al. [18]	Finland	44	81	50 (12 months)
Ryan et al. [19]	United States	1,427	58	16 (during hospitalization)

To our knowledge, this is the first study examining seasonal variations in mortality associated with ASDH. While not specific to ASDH pathologies, several other studies have found similar excess in winter mortality across a range of disease states: In an analysis of emergency hospital admissions, Hajat et al. reported a 3.4% increase in all deaths and a 0.78% increase in emergency admissions for every 1 °C drop in temperature [20]. Prospective work by Lorking et al. additionally found winter excess in stroke-related hospitalization, length of stay, and mortality using a tropical region patient population, results of which were repeated in other global regions such as the United Kingdom in the study done by Myint et al. [21,22]. Finally, a study conducted by Callaly et al. found the minimum daily temperature to be independently associated with a 20% increase in in-hospital mortality among elderly emergency admissions using a 10-year retrospective database [23]. Possible explanations for increased winter mortality from both ASDH and other traumatic injuries in elderly patients include traumatic falls as a result of icy conditions, as well as increased travel time from the site of injury to the hospital in the setting of adverse outdoor conditions. Time to treatment in particular has been reported as one of the major predictive factors for mortality in ASDH [24] although this finding has not been shown in other studies [25].

Previous authors have identified age as an independent predictor of mortality after surgery in ASDH [26]. With our current investigation reporting a study cohort with a mean age of 70.4, we surmise that our findings of high mortality associated with ASDH in the winter months may be closely tied to the epidemiology of the elderly population suffering from ASDH. The incidence of ASDH among the elderly population is rising, with falls in the elderly equaling and even surpassing motor vehicle accidents as the most frequent cause of TBI and ASDH [2,27]. Injuries among elderly individuals occur more frequently in the winter due to increased environmental hazards. Falls and fractures among the elderly reportedly increase fivefold in months with ice and snow [28]. Elderly patients are also at high risk of sustaining negative outcomes due to increased frailty, a phenomenon of decreased physiological reserve to accommodate biological stressors [29]. A higher prevalence of comorbid conditions and the use of anticoagulants also can increase the risk of complications, leading to higher mortality in elderly patients [8]. Elderly patients with TBI have worse mortality and functional outcomes after surgery [4,18] and during discharge [30].

There are several limitations in this study. The retrospective nature of our data collection is subject to reporting and missing data biases. Additionally, the original design of NSQIP may not have been set up to answer this particular question. The ACS NSQIP database only reports admissions of patients in three-month periods. Our study is, therefore, restrained in defining winter as January, February, and March (Quarter 1). The cross-sectional design of this study precludes establishing any causal relationship between winter and mortality due to ASDH. Unfortunately, we were unable to conclusively demonstrate a geographic component to this mortality. Considering the seasonal variations in different regions of North America, it would have been logical to discover that mortality was worse in states with predominately lower temperatures. However, to submit data to NSQIP by the Hospital Participation Agreement (HPA), all identifiers, including geographic information, are removed. NSQIP makes significant efforts to prevent the identification of hospitals, healthcare providers, or patients, and this limitation constrained the scope of the current investigation.

Notwithstanding these limitations, our study is the first to investigate the seasonal variation of mortality due to ASDH using a nationally representative sample of the U.S. population. NSQIP also adjusts for confounding variables such as the complexity of surgical procedures and the extent of the patient's illness. Given that we examined a population affected by traumatic injury, there is a significant risk of confounding bias from potential poly-trauma. Although our study was not designed to adjust for these confounding variables, repeating this work to identify independent predictors is suggested. 

Our study indicates a trend of increased ASDH mortality during the winter that, combined with additional studies, may aid in informing the management decisions of healthcare practitioners. We hope that this publication inspires more inspection into our delivery care model and the need to improve the emergency system, especially at particularly challenging times of the year. We anticipate innovations in our healthcare system that strive to bring the highest possible level of care to patients and that may very well include increasing the number of first-care responders in the first quarter of the year to meet the needs discovered in this investigation.

## Conclusions

In this study, the incidence of mortality in patients admitted due to ASDH was found to be significantly greater in the winter months of January, February, and March when compared to other months. Further exploration of the relationship between seasonal changes, outdoor temperatures, and traumatic injury such as ASDH is warranted. Additional investigation into seasonal variation among epidural hematoma patients and other traumatic pathologies in the elderly population is also likely to provide greater insight into the unique risk factors encountered by this population. Finally, elucidation of the underlying causes of our reported findings may inform patterns in acute care for future clinical decision-making.
